# Editorial: Nutrition and sustainable development goal 5: gender equality

**DOI:** 10.3389/fnut.2024.1384066

**Published:** 2024-02-27

**Authors:** Romanus Osabohien, Oluwatoyin Matthew

**Affiliations:** ^1^Institute of Energy Policy and Research (IEPRe), Universiti Tenaga Nasional (UNITEN), Kajang, Malaysia; ^2^University of Religions and Denominations, Qom, Iran; ^3^DePECOS Institutions and Development Research Centre (DIaDeRC), Ota, Nigeria; ^4^Department of Economics and Development Studies, Covenant University, Ota, Nigeria

**Keywords:** food and nutrition security, gender equality, sustainable development goals, SDG2: zero hunger, malnutrition

## Introduction

The connection between nutrition and gender equality lies at the heart of sustainable development efforts, encapsulated within the transformative goals of the 2030 Agenda for Sustainable Development. Sustainable Development Goal 5 (SDG 5) specifically focuses on achieving gender equality and empowering all women and girls, highlighting the imperative to address disparities and promote inclusivity across various spheres, including nutrition (1).

Women, particularly in low- and middle-income countries, often confront unique nutritional challenges rooted in sociocultural norms, limited access to resources, and discriminatory practices that impact their dietary diversity and overall wellbeing (2–4). Studies have shown that gender disparities in nutrition significantly affect women's health, with implications extending to maternal and child health outcomes (5). As such, addressing gender-specific nutritional needs holds a crucial place within the broader framework of achieving gender equality and fostering sustainable development.

This guest editorial seeks to unpack and critically examine the multifaceted relationship between nutrition and gender equality, delving into the unique challenges faced by women in accessing nutritious diets, the impact of gender-responsive nutrition initiatives, and their resonance with the principles enshrined in SDG 5. By exploring the gendered dimensions of nutrition and its pivotal role in advancing gender equality, this editorial endeavors to stimulate constructive dialogues and policy actions that propel the synergistic pursuits of nutrition security and gender parity within the global sustainable development landscape. The studies collated in this Research Topic are summarized in the next section.

## Summary of studies in the Research Topic

Five studies included in the Research Topic are summarized in this section. The first study, conducted by Wakwoya et al., is titled *Determinants of nutritional status among pregnant women in East Shoa Zone, Central Ethiopia*. This study aimed to determine the level of undernutrition and identify factors associated with undernutrition among pregnant women attending public health facilities in the East Shoa Zone, Central Ethiopia. The findings show that the prevalence of undernutrition among pregnant women was 13.9%. On the other hand, women's decision-making power and nutritional counseling were independently associated with the nutritional status of pregnant women. The findings indicate that a significant number of pregnant women in the study were undernourished.

The second study, conducted by Mechlowitz et al., is titled *Women's empowerment and child nutrition in a context of shifting livelihoods in Eastern Oromia, Ethiopia*. This study used randomly selected mothers of children aged 10–15 months from Haramaya district, Eastern Hararghe, Oromia, Ethiopia, as survey participants and applied the nested logistic regression models. The results indicate that production and tropical livestock units were not significantly associated with any of the child nutrition outcomes. Furthermore, the third study, conducted by Tsai et al., explores the association between a vegetarian diet and varicose veins, a condition that might be more prevalent in men than in women. The study involved 9,905 adults whose data were obtained from Taiwan Biobank between 2008 and 2020. The findings show that women were more susceptible to varicose veins compared to men, regardless of diet. However, in terms of diet, only men who followed a vegetarian diet were at a higher risk of developing VVs.

The fourth study, conducted by Kara et al., is titled *Factors Associated with Inadequate Dietary Diversity among Adolescent Girls in Hurumu Woreda High School, Oromia Region, Southwest, Ethiopia*. The study used an institution-based cross-sectional design, involving 374 high school-age adolescent girls and applied binary and multivariable logistic regressions for analysis. The findings show that the magnitude of inadequate dietary diversity among adolescent girls in this study was 62.6%. Living with more than five family members, consumption of sweet foods/beverages, poor nutritional knowledge, and poor household wealth were significantly associated with inadequate dietary diversity.

Living with more than five family members, poor household wealth status, consumption of sweet foods/beverages, family size, and poor nutritional knowledge were factors significantly associated with inadequate dietary diversity. Hence, it is recommended to implement nutrition education programs, promote the use of family planning methods, and create opportunities for securing income-generating activities. The fifth study, conducted by Deslippe et al., is titled *Boys and girls differ in their rationale behind eating: a systematic review of intrinsic and extrinsic motivations in dietary habits across countries*. The study engaged four databases that were searched following the PRISMA guidelines. The study concluded that the food habits of boys and girls are not motivated by the same factors. To create more effective dietary interventions targeting health promotion, the unique motivations behind food habits need to be understood and incorporated.

## Conclusion

The intricate connections between nutrition and Sustainable Development Goal 5 represent a critical frontier in the pursuit of gender equality and sustainable development. Recognizing the gendered dimensions of nutrition and addressing the nuanced nutritional needs of women are fundamental to achieving inclusive and equitable progress. Efforts aimed at enhancing women's nutritional wellbeing and promoting gender-responsive nutrition initiatives are vital for realizing the transformative objectives of SDG 5. As we navigate the complex terrain of sustainable development, it is essential to uphold the principles of gender equality and prioritize gender-responsive nutritional interventions as integral components of broader strategies to ensure sustainable and equitable development for all.

## Author contributions

RO: Conceptualization, Data curation, Methodology, Writing—original draft, Writing—review & editing. OM: Supervision, Writing—original draft, Writing—review & editing.
